# Temporal Patterns of Vertigo and Migraine in Vestibular Migraine

**DOI:** 10.3389/fnins.2020.00341

**Published:** 2020-04-15

**Authors:** Manyun Yan, Xiaoning Guo, Wei Liu, Jiajie Lu, Jingwen Wang, Lan Hu, Kaijian Xia, Jianqiang Ni, Haifeng Lu, Hongru Zhao

**Affiliations:** ^1^Department of Neurology, The First Affiliated Hospital of Soochow University, Suzhou, China; ^2^Department of Neurology, Ninth People’s Hospital, Suzhou, China; ^3^Changshu No. 1 People’s Hospital, Suzhou, China

**Keywords:** vestibular migraine, temporal patterns, vestibular symptoms, migraine features, interaction

## Abstract

Vestibular migraine (VM) is a multidisciplinary disease under exploration. Multiple temporal patterns of vertigo and migraine make it difficult to diagnose VM, and their effect on the clinical features of VM is still unclear. Here we investigated the clinical features of VM under three temporal patterns. 172 VM patients were enrolled in this study and divided into three groups: 86 patients in group A had an earlier onset of migraine than vertigo, 35 patients in group B had an earlier onset of vertigo than migraine, and 51 patients in group C had concurrent vertigo and migraine. No significant difference was found among three groups regarding types, intensity and accompanying symptoms of the vestibular attack. Patients in group C presented higher frequency and longer duration of vertigo than group A and B, while patients in group A presented lower frequency and shorter duration of headaches than group B and C. Additionally, the frequency, duration, intensity and accompanying symptoms of headache in group A decreased significantly after the onset of vertigo, especially in women around menopause. We hypothesized that vestibular stimulation could inhibit the trigeminal pain pathway, while painful trigeminal stimulation could excite the vestibular system. Our findings may contribute to the clinical identification of VM and further clarification of its pathogenesis.

## Introduction

Vertigo and migraine are both very common complaints among patients, and a strong link between them has been proved. The co-occurrence of migraine and vertigo in the same individual was expected in 1% of the population based on the prevalence of migraine and vestibular vertigo, but the actual percentage was about three times higher than expected (Neuhauser et al., 2006). Moreover, migraine patients had a two- or three-fold higher risk for vertigo than those without headache (Vukovic et al., 2007). In 1999, Dieterich and Brandt introduced the term “vestibular migraine” (VM) to describe the clinical condition that associated with vestibular symptoms and migraine headache (Dieterich and Brandt, 1999). In recent years, VM, including definite VM and probable VM, has been considered as an independent diagnostic entity and one of the most common causes of vertigo (Lempert et al., 2012; Arnold, 2018; Huang et al., 2020).

However, VM has been remained underdiagnosed despite ongoing studies in recent years. Only 8–20% of VM patients were correctly diagnosed in practice (Neuhauser et al., 2006; Geser and Straumann, 2012; Formeister et al., 2018). A big challenge for diagnosis is multiple temporal patterns of vertigo and migraine in VM. 51–65.6% of VM patients suffered from migraine before the onset of vertigo (Morganti et al., 2016; Zhang et al., 2016; Beh et al., 2019), and there was an interval of 8–20 years between the two symptoms (Cohen et al., 2011; Pagnini et al., 2014). 10.2–13% of patients had an earlier onset of vertigo than migraine (Qiu et al., 2014; Zhang et al., 2016). 34–49.6% of patients had migraine headaches during vestibular attacks (Zhang et al., 2016; Beh et al., 2019). Since most of VM patients experienced vertigo separately from headache, and even some patients had vestibular symptoms years after the headache disappeared, they often did not actively report headache history. One study showed that only 9.8% of VM patients reported headache symptoms before being carefully asked (Ren et al., 2014). Likewise, clinicians often neglect to ask the history or associated symptoms of migraine in patients complaining with vertigo, which often results in misdiagnosis or missed diagnosis. Therefore, the investigation of temporal patterns of vertigo and migraine and their impact on patients with VM is very important for understanding and management of VM.

The aim of this study was to describe and compare the clinical features of VM patients with different temporal patterns, and investigate the interaction between vertigo and migraine headache in VM.

## Materials and Methods

### Participants

**FIGURE 1:**
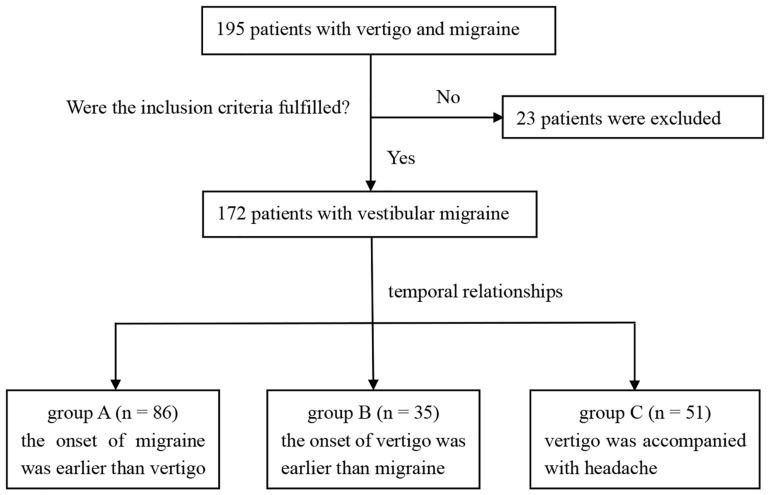
Flow diagram of participants’ selection.

Patients who met diagnostic criteria of definite VM (Arnold, 2018) or probable VM (Lempert et al., 2012) in neurology and headache clinics of First Affiliated Hospital of Soochow University from January 2018 to April 2019 were enrolled (Figure 1). The exclusion criteria were as follows: other causes of vestibular attack such as benign paroxysmal positional vertigo (BPPV), Meniere’s disease or transient ischemic attack of posterior circulation; history of head trauma; severe physical illness; abnormal computed tomography or magnetic resonance imaging (MRI); history of alcohol or drugs abuse; other primary or secondary headaches; dizziness associated with chronic anxiety; history of intracranial infection.

### Data Collection

A structured questionnaire was used to interview the participants regarding the following aspects: Demographics, familial history of headache or vertigo, age of onset of vertigo and migraine, vertigo intensity (Arnold, 2018) (moderate: vestibular symptoms interfere with but do not prevent daily activities; severe: daily activities cannot be continued), pain intensity [visual analog scale (VAS)], frequency (number of attacks within 3 months before enrolled), duration (minutes to hours), accompanying symptoms of vertigo (nausea, vomiting, phonophobia, photophobia, tinnitus), accompanying symptoms of headache (nausea, vomiting, phonophobia, photophobia, osmophobia, neck stiffness, scalp allodynia). Vestibular attacks included spontaneous vertigo (internal or external vertigo), positional vertigo (occurring after a change of head position), visually induced vertigo (triggered by a complex or large moving visual stimulus), head motion-induced vertigo (occurring during head motion), head motion-induced dizziness with nausea (dizziness is characterized by a sensation of disturbed spatial orientation) (Arnold, 2018). Furthermore, psychiatric comorbid disorders [anxiety, depression, persistent postural-perceptual dizziness (PPPD) (Staab et al., 2017) and sleep disorders] were assessed at enrollment.

We defined the temporal patterns between migraine and vertigo as follows: (A) the onset of migraine was earlier than vertigo: (i) between migraine and vertigo there was a symptom-free interval (at least one year), (ii) migraine shifted directly into vertigo without a free interval (iii) migraine gradually changed into vertigo; (B) the onset of vertigo was earlier than migraine; (C) vertigo was accompanied with headache. Moreover, headache features before the onset of vertigo in group A were additionally collected, including frequency, duration, intensity and accompanying symptoms.

### Statistical Analyses

All statistical analyses were performed in SPSS software version 22.0. Categorical variables were compared using Chi-square test and presented as frequency counts and percentages. As all continuous variables in this study were non-normal distribution, they were presented as the median and interquartile range, and the Mann–Whitney *U* test was used for comparison between two groups or the Kruskal–Wails H test among three groups. Statistically significance was set at a two-sided *p*-value < 0.05.

## Results

**FIGURE 2:**
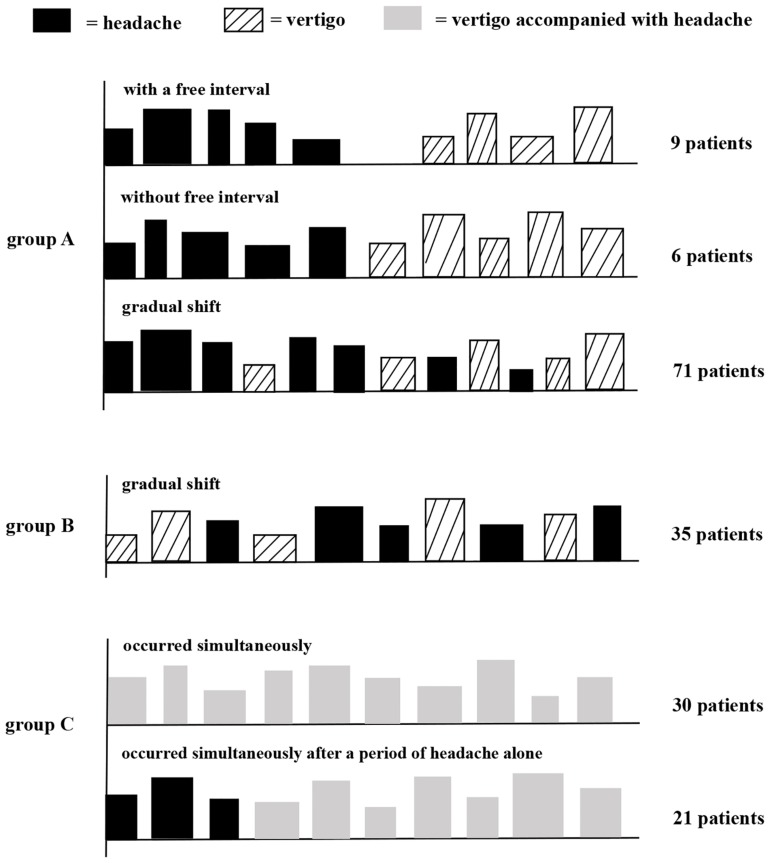
Schematic diagram of temporal patterns of vertigo and migraine in VM patients. Group A, patients had an earlier onset of migraine than vertigo; group B, patients had an earlier onset of vertigo than migraine; and group C, patients had concurrent vertigo and migraine. Height and width of rectangles indicate, respectively, the strength and duration of headache or vertigo.

172 VM patients were enrolled in this study and divided into three groups based on the temporal patterns of vertigo and migraine as shown in Figure 2. In group A, 86 patients (50.0%) had an earlier onset of migraine than vertigo. Nine patients (5.2%) reported vertigo attacks after the migraine headaches disappeared, with a symptom-free interval of 1–10 years. Six patients (3.5%) reported that vertigo occurred immediately after the disappearance of headaches without any interval. 71 cases (41.3%) reported the gradual shift of the two symptoms, with a partial overlap. In group B, 35 patients (20.3%) had an earlier onset of vertigo than migraine. In group C, 51 patients (29.7%) had concurrent vertigo and migraine headache. Among them, thirty patients (17.4%) initially presented with vertigo and headache simultaneously, and 21 patients (12.2%) complained that vertigo occurred together with headache after a period of migraine headache attacks alone.

Three groups were female-dominated, but more female subjects in group A developed vertigo around menopause than in group B (*p* < 0.05) and C (*p* = 0.084). Patients in group B presented a lower age of onset of vertigo compared to group A and C (*p* < 0.01) as expected. Patients in group A presented a lower age of onset of migraine compared to group B (*p* < 0.05), but similar age of onset to group C [*p*: not significant (NS)]. The proportion of familial history of migraine in group B was lower than that in group A and C (*p* < 0.05). The proportion of motion sickness history in group C was higher than that in group B (*p* < 0.05) (Table 1).

**TABLE 1:** Demographic data among subjects in group A, B, and C.

	**A (*n* = 86)**	**B (*n* = 35)**	**C (*n* = 51)**
Age at inclusion (years)	49 (37, 59)	43 (33, 60)	47 (33, 57)
Sex (female %)	69 (80%)	30 (86%)	41 (80%)
Vertigo occurred around menopause (of females)	28 (41%)	5 (17%)*	10 (24%)
Age of onset of vertigo (years)	43 (33, 54)	29 (22, 37)*	40 (30, 49)†
Age of onset of migraine (years)	32 (25, 41)	37 (31, 42)*	32 (25, 43)
Familial history of vertigo	21 (24%)	9 (26%)	18 (35%)
Familial history of migraine	36 (42%)	7 (20%)*	21 (41%)†
Motion sickness history	50 (58%)	17 (49%)	37 (73%)†

A: the onset of migraine was earlier than vertigo; B: the onset of vertigo was earlier than migraine; C: vertigo was accompanied with headache. *p < 0.05 vs. group A; †*p* < 0.05 vs. group B.

No significant difference was found among groups regarding types, intensity and accompanying symptoms of vestibular attack (*p*: NS). Patients in group C presented higher frequency and longer duration of vertigo than group A and B. (Table 2).

**TABLE 2:** Vestibular symptoms among subjects in group A, B, and C.

**Vestibular symptoms**	**A (*n* = 86)**	**B (*n* = 35)**	**C (*n* = 51)**
**Vestibular attack**			
Spontaneous vertigo	38 (44%)	14 (40%)	15 (29%)
Positional vertigo	26 (30%)	11 (31%)	15 (29%)
Head motion dizziness with nausea	24 (28%)	12 (34%)	22 (43%)
Head-motion vertigo or visual vertigo	4 (5%)	1 (3%)	1 (2%)
Frequency (times/3 months)	3 (1, 9)	3 (0.6, 10.5)	6 (3, 12)*†
Duration (h)	2 (0.3, 18.5)	2.5 (0.5, 30)	18 (2.5, 24)*
5 min–1 h	33 (38%)	14 (40%)	9 (18%)*†
1–24 h	38 (44%)	13 (37%)	25 (49%)
24–72 h	15 (17%)	8 (23%)	17 (33%)*
Intensity (severe %)	33 (38%)	11 (31%)	13 (26%)
**Accompanying symptoms**			
Nausea	61 (71%)	20 (57%)	37 (73%)
Vomiting	51 (59%)	17 (49%)	26 (51%)
Photophobia	42 (49%)	18 (51%)	21 (41%)
Phonophobia	57 (66%)	23 (66%)	33 (65%)
Tinnitus	20 (23%)	6 (17%)	11 (22%)

A: the onset of migraine was earlier than vertigo; B: the onset of vertigo was earlier than migraine; C: vertigo was accompanied with headache. *p < 0.05 vs. group A; †*p* < 0.05 vs. group B.

Patients in group A presented lower frequency and shorter duration of migraine headaches than group B and C (*p* < 0.05) (Table 3). Furthermore, the frequency, duration, intensity and accompanying symptoms of headache in group A decreased significantly after the onset of vertigo (Table 4). Patients in group C presented stronger intensity of headache and reported nausea and vomiting during headache attacks more often compared to patients in group A (*p* < 0.001) and B (*p* < 0.05) (Table 3).

**TABLE 3:** Headache symptoms among subjects in group A, B, and C.

**Headache symptoms**	**A (*n* = 86)**	**B (*n* = 35)**	**C (*n* = 51)**
Frequency (times/3 months)	3 (0.3, 7.9)	6 (3, 12)*	7.5 (3, 16.5)*
Duration (h)	12 (2, 24)	24 (5, 36)*	24 (4.5, 36)*
Intensity (VAS)	5 (3, 6)	5 (4, 7)	5 (5, 7)*†
**Accompanying symptoms**			
Nausea	34 (40%)	18 (51%)	38 (75%)*†
Vomiting	20 (23%)	10 (29%)	27 (53%)*†
Photophobia	26 (30%)	17 (49%)	21 (41%)
Phonophobia	47 (55%)	23 (66%)	35 (69%)
Osmophobia	4 (5%)	4 (11%)	6 (12%)
Neck stiffness	13 (15%)	9 (26%)	14 (27%)
Scalp allodynia	14 (16%)	5 (14%)	5 (10%)

A: the onset of migraine was earlier than vertigo; B: the onset of vertigo was earlier than migraine; C: vertigo was accompanied with headache. VAS: visual analog scale. *p < 0.05 vs. group A; †*p* < 0.05 vs. group B.

**TABLE 4:** Headache symptoms before and after the onset of vertigo in group A.

**Headache symptoms**	**Before (*n* = 86)**	**After (*n* = 86)**	***p-*value**
Frequency (times/3 months)	6 (3, 9)	3 (0, 8)	0.015
Duration (h)	24 (7.5, 36)	12 (2, 24)	0.001
Intensity (VAS score)	6 (5, 7)	5 (3, 6)	< 0.001
**Accompanying symptoms**			
Nausea	44 (51%)	34 (40%)	0.126
Vomiting	29 (34%)	20 (23%)	0.128
Photophobia	38 (44%)	26 (30%)	0.058
Phonophobia	60 (70%)	47 (55%)	0.041
Osmophobia	8 (9%)	4 (5%)	0.369
Neck stiffness	23 (27%)	13 (15%)	0.061
Scalp allodynia	22 (26%)	14 (16%)	0.134
Number of accompanying symptoms	2 (2, 4)	2 (0, 3)	0.002

*Group A: the onset of migraine was earlier than vertigo; VAS: visual analog scale.*

Following the onset of vestibular or headache symptoms, many were diagnosed with anxiety (36.6%), depression (47.7%), PPPD (34.9%) and sleep disorders (70.3%). Except that the proportion of anxiety in group C was higher than that in group A (*p* < 0.05), no significant difference in the proportion of psychiatric comorbid disorders was found among groups (*p*: NS) (Table 5).

**TABLE 5:** Psychiatric comorbid disorders among subjects in group A, B, and C.

**Disorders**	**A (*n* = 86)**	**B (*n* = 35)**	**C (*n* = 51)**
Anxiety	24 (28%)	13 (37%)	26 (51%)*
Depression	40 (47%)	15 (43%)	27 (53%)
PPPD	30 (35%)	15 (43%)	15 (29%)
Sleep disorders	56 (65%)	29 (83%)	36 (71%)

A: the onset of migraine was earlier than vertigo; B: the onset of vertigo was earlier than migraine; C: vertigo was accompanied with headache. PPPD: persistent postural-perceptual dizziness. *p < 0.05 vs. group A.

## Discussion

Temporal patterns of vertigo and migraine in VM patients and how they affect the clinical features of VM have not yet been systematically described. In this study, we investigated the clinical features of VM under three temporal patterns and found that patients with vertigo and migraine occurred simultaneously presented higher frequency and longer duration of vertigo, while patients with earlier onset of migraine than vertigo presented lower frequency and shorter duration of headache.

Previous studies have suggested that headaches significantly decreased in frequency and strength or disappeared after the onset of vertigo in VM patients (von Brevern and Lempert, 2016; Teggi et al., 2018). This phenomenon was more common in women around menopause (Lempert et al., 2009; Park and Viirre, 2010). A 13-year observational study showed that headaches completely ceased after vertigo attacked in 57% of patients and substantially improved in 43% (Pagnini et al., 2014). However, there were no detailed data to clarify the specific changes in migraine headache features before and after the onset of vertigo. In our cases, we noticed a 50% reduction in frequency and duration of headache after the onset of vertigo, a 17% reduction in pain intensity and a 22–50% reduction in accompanying symptoms such as nausea, vomiting, photophobia, phonophobia, osmophobia, neck stiffness, and scalp allodynia. The difference between our study and the previous study (Pagnini et al., 2014) was that only 19 patients (22%) reported complete cessation of headache in this study, 35% noticed a considerable reduction in frequency of headache after vertigo, 23% got relief for duration, intensity or accompanying symptoms, but in 20% cases, headache symptoms were unaffected by vertigo, which was partly due to the different inclusion criteria. We included all patients diagnosed with VM, and the previous study (Pagnini et al., 2014) only included patients who developed vestibular symptoms after the disappearance or remarkable reduction of headache. Another reason was that this study was not a longitudinal study, and there was recall bias in the features of migraine headache prior to the onset of vertigo.

The mechanisms of disappearance or attenuation of headache after recurrent vertigo in VM are unclear. We hypothesized that one potential mechanism was the interaction of the vestibular and trigeminal systems. The main mechanism of VM is currently considered as the connection of trigeminal caudal nucleus with vestibular nucleus (Furman et al., 2013; Huang et al., 2020), so the vestibular stimulation and migraine headache could interact with each other. A study showed that headaches completely disappeared or reduced in strength after vestibular thermal stimulation during migraine attacks (Kolev, 1990), which suggested that the painful conduction pathway might be inhibited after the activation of the vestibular pathway. On the other hand, both the prevalence of migraine and VM was significantly higher in females than males, and the conversion from headache to vertigo was more common around menopause (Lempert et al., 2009; Park and Viirre, 2010; Pagnini et al., 2014), which has been confirmed in our data. Moreover, migraine tended to improve when sex hormone levels stabilized after menopause (Vetvik and MacGregor, 2017), and hormone fluctuations during the perimenopausal period were associated with an increased risk of vertigo (Park and Viirre, 2010). Hence, another possible hypothesis was the role of female sex hormones as a facilitating factor for the transformation of migraine headaches into vestibular attacks.

An interesting aspect verified in our study was that VM patients with vertigo and headache occurred simultaneously presented higher frequency and longer duration of vertigo. Similarly, this phenomenon might be due to the interaction of the vestibular and trigeminal systems. A study showed that trigeminal stimulation induced nystagmus in patients with migraine but not controls, which suggested increased vestibular excitability in migraine patients (Marano et al., 2005). Functional imaging of the brain showed that the activation of temporo-parieto-insular areas and bilateral thalami was increased during vertigo attacks in VM patients (Shin et al., 2014), and the magnitude of thalamic activation was positively correlated with the frequency of migraine attacks (Russo et al., 2014). Based on this, we hypothesized that vestibular excitability increased when vertigo occurred together with migraine headaches, and the thalamus and other brain regions might be activated more significantly after both vestibular and painful stimulation. That is to say, painful trigeminal stimulation might enhance the excitability of the vestibular system, and the threshold of vestibular perception of VM patients decreases and the sensitivity increases, similar to the central sensitization effect. In addition, patients with concurrent vertigo and headache experienced severer headaches accompanied with nausea and vomiting more often, which might be associated with more frequent attacks of vertigo. But no significant correlations were found between other clinical features of migraine (e.g., disease duration, pain intensity, migraine disability) and thalamic activation, except for the frequency (Russo et al., 2014). And the influence of migraine symptoms on vestibular attacks remains to be further studied. Other factors that should be considered were the contributions of motion sickness history and anxiety, which were more common in VM patients with concurrent vertigo and migraine. Anxiety was associated with significantly increased recurrence of VM (Formeister et al., 2018) and motion sickness could enhance motion intolerance (Wang and Lewis, 2016).

The special temporal patterns of vertigo and migraine make it difficult for many clinicians to accurately diagnose VM, and patients usually undergo a painful and protracted course until they visit a headache or vertigo specialist. During the course, VM patients are usually anxious about unexpected and intense vertigo attacks, afraid of falling and avoid going out or entering various environments triggering dizziness (Kutay et al., 2017). Additionally, many of them complain of decreased daily activities and nearly constant dizziness and lightheaded (von Brevern and Lempert, 2016). Many studies suggested that VM patients were prone to psychiatric comorbidities, such as anxiety (19.8–70.2%) (Langhagen et al., 2014; Beh et al., 2019), depression (21.8–40.5%) (Vuralli et al., 2018; Beh et al., 2019), PPPD (32.8–41%) (Neff et al., 2012; Eggers et al., 2014; Beh et al., 2019) and sleep disorders (29–67.4%) (Vuralli et al., 2018; Beh et al., 2019; Wu et al., 2019), regardless of the frequency of vertigo. Our study showed that VM patients also had high rates of psychiatric comorbidities regardless of the temporal patterns. Vestibular attacks increase the incidence of psychiatric comorbidities, and psychiatric disorders aggravate the severity of vestibular symptoms. Then a vicious circle is formed, and the disease becomes deferred. Therefore, we should make an effort to understand these temporal patterns in VM, in order to give patients correct diagnosis and timely treatment.

This study had several potential limitations. Firstly, we lacked data on the features of vertigo before the onset of migraine in group B to further support our hypothesis that migraine headaches could increase the vestibular excitability and aggravate the manifestations of vertigo. Moreover, we only described the clinical features of VM by a cross-sectional study. A long-term longitudinal study will be of great help to understand the temporal patterns of VM and identify this entity. Secondly, we enrolled patients with probable VM, the diagnostic criteria of which have not been included in the International Classification of Headache Disorders 3rd edition (ICHD-3). But some studies have shown that there was no significant difference between definite and probable VM (Eggers et al., 2011; Van Ombergen et al., 2015; Cho et al., 2016), and most probable VM patients would develop definite VM over time during long-term follow up (Radtke et al., 2011).

## Conclusion

We found that the temporal patterns of vertigo and migraine affected the clinical features of VM. Migraine headaches usually disappeared or relieved after recurrent vertigo attacks, especially in women around menopause, while vestibular vertigo was more frequent and lasted longer when accompanied with migraine headaches. The underlying mechanism might be that vestibular stimulation inhibits the trigeminal pain pathway, while painful trigeminal stimulation could excite the vestibular system.

## Data Availability Statement

All datasets generated for this study are included in the article/supplementary material.

## Ethics Statement

The studies involving human participants were reviewed and approved by the Institutional Review Board of The First Affiliated Hospital of Soochow University. The patients/participants provided their written informed consent to participate in this study.

## Author Contributions

HZ, HL, JN, and MY designed the study. HZ, MY, XG, WL, JL, LH, JW, and KX evaluated the subjects and collected the data. MY and XG analyzed the data. MY wrote the initial draft, with HL and HZ participating in revising the manuscript.

## Conflict of Interest

The authors declare that the research was conducted in the absence of any commercial or financial relationships that could be construed as a potential conflict of interest.
